# 3′ RNA Uridylation in Epitranscriptomics, Gene Regulation, and Disease

**DOI:** 10.3389/fmolb.2018.00061

**Published:** 2018-07-13

**Authors:** Miriam R. Menezes, Julien Balzeau, John P. Hagan

**Affiliations:** Department of Neurosurgery, University of Texas Health Science Center at Houston, Houston, TX, United States

**Keywords:** RNA epitranscritpomics, 3′ terminal RNA uridylation, TUTase, LIN28/let-7 pathway, DIS3L2, cancer, perlman syndrome, Wilms tumor

## Abstract

Emerging evidence implicates a wide range of post-transcriptional RNA modifications that play crucial roles in fundamental biological processes including regulating gene expression. Collectively, they are known as epitranscriptomics. Recent studies implicate 3′ RNA uridylation, the non-templated addition of uridine(s) to the terminal end of RNA, as a key player in epitranscriptomics. In this review, we describe the functional roles and significance of 3′ terminal RNA uridylation that has diverse functions in regulating both mRNAs and non-coding RNAs. In mammals, three Terminal Uridylyl Transferases (TUTases) are primarily responsible for 3′ RNA uridylation. These enzymes are also referred to as polyU polymerases. TUTase 1 (TUT1) is implicated in U6 snRNA maturation via uridylation. The TUTases TUT4 and/or TUT7 are the predominant mediators of all other cellular uridylation. Terminal uridylation promotes turnover for many polyadenylated mRNAs, replication-dependent histone mRNAs that lack polyA-tails, and aberrant structured noncoding RNAs. In addition, uridylation regulates biogenesis of a subset of microRNAs and generates isomiRs, sequent variant microRNAs that have altered function in specific cases. For example, the RNA binding protein and proto-oncogene LIN28A and TUT4 work together to polyuridylate pre-let-7, thereby blocking biogenesis and function of the tumor suppressor let-7 microRNA family. In contrast, monouridylation of Group II pre-miRNAs creates an optimal 3′ overhang that promotes recognition and subsequent cleavage by the Dicer-TRBP complex that then yields the mature microRNA. Also, uridylation may play a role in non-canonical microRNA biogenesis. The overall significance of 3′ RNA uridylation is discussed with an emphasis on mammalian development, gene regulation, and disease, including cancer and Perlman syndrome. We also introduce recent changes to the HUGO-approved gene names for multiple terminal nucleotidyl transferases that affects in part TUTase nomenclature (TUT1/TENT1, TENT2/PAPD4/GLD2, TUT4/ZCCHC11/TENT3A, TUT7/ZCCHC6/TENT3B, TENT4A/PAPD7, TENT4B/PAPD5, TENT5A/FAM46A, TENT5B/FAM46B, TENT5C/FAM46C, TENT5D/FAM46D, MTPAP/TENT6/PAPD1).

## Introduction

Epitranscriptomics refer to a diverse set of RNA chemical modifications and post-transcriptional nucleotide additions that play central roles in pre-mRNA splicing, translation, and regulation of gene expression. For example, ribosomal and transfer RNAs undergo extensive chemical modifications that are required for function through stabilization of their RNA secondary structure, while non-templated nucleotide additions are critical for polyadenylated mRNAs and aminoacylation of tRNAs. Recent evidence implicates 3′ RNA uridylation, the addition of non-templated uridine(s) to the RNA end, in several biological processes. This review presents our current understanding of the biochemical and functional roles for 3′ terminal RNA uridylation with a focus on mammalian biology. Over the past decade, 3′ RNA uridylation has emerged to be functionally significant for multiple RNA types such as mRNAs, microRNAs, and structured non-coding RNAs. Terminal Uridylyl Transferase (TUTases) that are also known as polyU polymerases are the enzymes responsible for 3′ RNA uridylation.

## Non-canonical terminal ribonucleotidyl transferases

**Figure 1:**
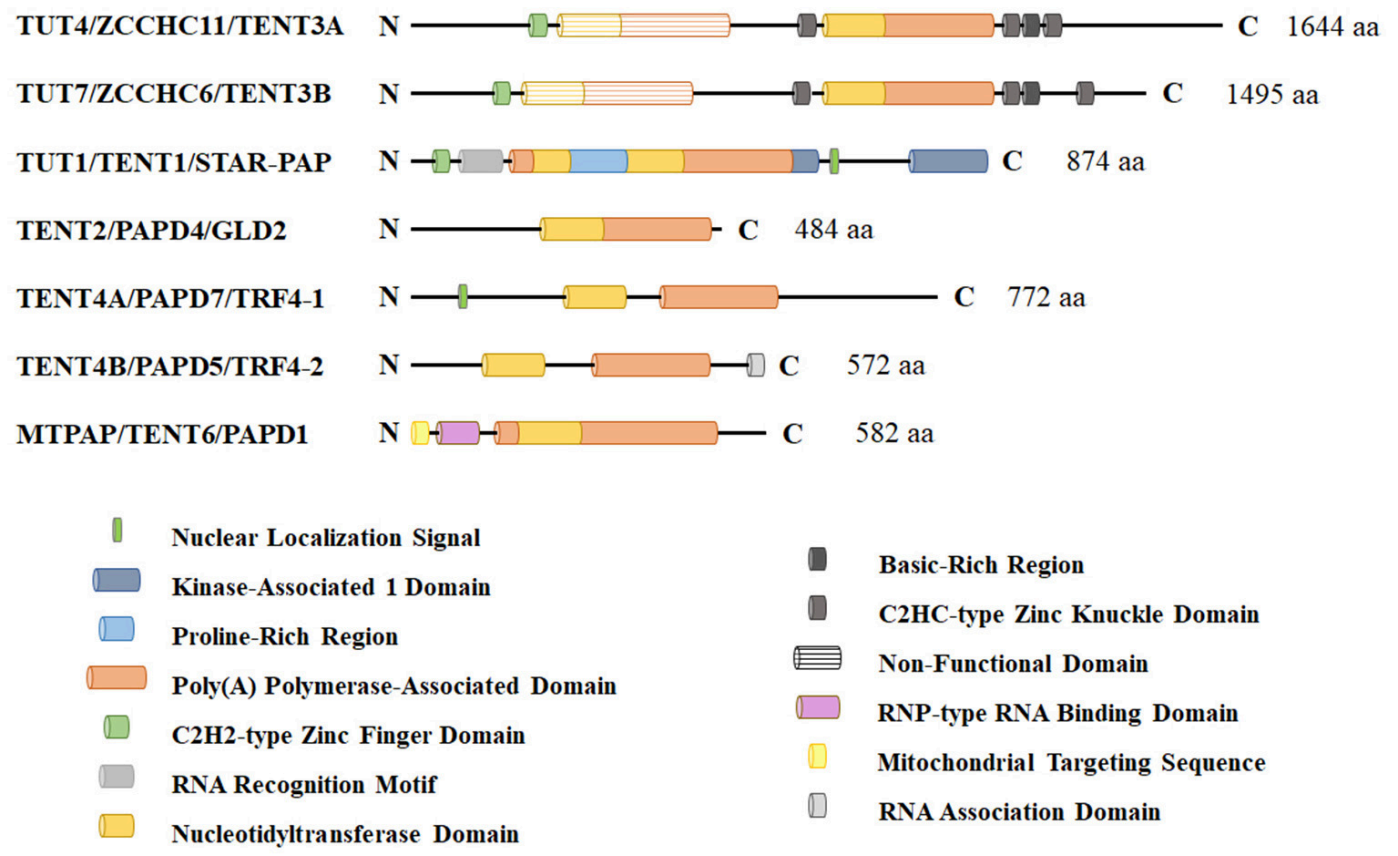
Schematic representation of non-canonical terminal ribonucleotdyl transferases in humans. These enzymes contain a DNA polymerase β-like nucleotidyltransferase domain and a more C-terminal polyA polymerase-like domain that is required for enzymatic activity. TUT1, TUT4, and TUT7 are the primary polymerases responsible for terminal 3′ RNA uridylation in mammalian cells. Other family members lack a critical histidine that confers UTP specificity and are thought to be primarily adenyltransferases. TUT1 has a nuclear localization signal and functions in part in nuclear U6 snRNA maturation and recycling. TUT4 and TUT7 are predominantly cytoplasmic proteins where they function in noncoding RNA quality control, mRNA turnover (both polyadenylated mRNAs and histone mRNAs), and regulation of let-7 microRNA biogenesis with LIN28A.

TUTases fall within a class of seven non-canonical terminal ribouncleotidyl transferases that contain a DNA polymerase β-like nucleotidyltransferase domain (Figure 1). Unfortunately, each member of the non-canonical polymerase family that includes TUTases has multiple names, creating considerable confusion in relation to their ribonucleotidyl specificity (Table 1). Here, we will refer to each enzyme using their HUGO-approved nomenclature that was recently updated by the Human Gene Nomenclature Committee to address several concerns. Two genes TUT4 (aka ZCCHC11) and TUT7 (aka ZCCHC6) encode terminal uridylyl transferases that are primarily cytoplasmic and are quite similar structurally, having arisen from a gene duplication event. These enzymes interact transiently with RNA and are slow polymerases (e.g., the TUT4 uridylation rate is ~0.2 nucleotides per second; Yeom et al., 2011). As such, they typically add one or very few uridines to their RNA substrates, except in cases where TUTase interaction with RNA is stabilized by other factors such as the RNA binding protein LIN28A. TUT1/STAR-PAP has dual specificity for UTP and ATP with respective *k*_cat_ values of 0.059 and 0.002 s^−1^ (Yamashita et al., 2017). The remaining non-canonical transferases primarily add non-templated adenosines. Phylogenetic and biochemical analyses reveal that the insertion of a histidine at the active site alters the ribonucleotidyl specificity of the polymerase from ATP to UTP (Munoz-Tello et al., 2012; Yates et al., 2015; Chung et al., 2016; Yamashita et al., 2017). Consistent with this idea, human TENT2 (aka GLD2/TUT2) lacks this critical histidine and prefers ATP over UTP by >80-fold (Chung et al., 2016). Furthermore, human TENT2 mutated by histidine insertion within the active site converts the mutant protein to a TUTase. In this review, we describe in detail the functional roles of three mammalian TUTases, TUT1, TUT4, and TUT7. Human TUT4 and TUT7 have numerous roles in regulating both mRNAs and non-coding RNAs. TUT1 has dual functions where it is critical for uridylation and maturation of the spliceosomal U6 snRNA in the nucleus and in the adenylation of select mRNAs, including HO-1 (Heme Oxygenase 1), BIK (BCL2-interacting killer), PTEN, WIF1, and CDH1 (Gonzales et al., 2008; Mellman et al., 2008; Li et al., 2012, 2017; Kandala et al., 2016; Sudheesh and Laishram, 2017). Lastly, we will discuss the roles of TUTases in mammalian development, physiology, and diseases including cancer and Perlman Syndrome.

**Table 1:** Nomenclature of Non-canonical terminal ribonucleotidyl transferases.

New HUGO-approved symbol*	**Approved name**	Old HUGO-approved symbol^*^	**Other alternate symbols**	**Subcellular localization**	**Disease**	**Ribonucleotide specificity**
TUT4	Terminal Uridylyl Transferase 4	ZCCHC11	TENT3A, PAPD3, KIAA0191	Cytoplasm (Heo et al., 2009; Schmidt et al., 2011) Nucleus>Cytoplasm (Minoda et al., 2006) Cytoplasm/Nucleolus (Barbe et al., 2008)	Numerous Cancer Types including breast, ovarian, and colon (Hallett and Hassell, 2011; Piskounova et al., 2011; Balzeau et al., 2017) Myotonic dystrophy (Rau et al., 2011)	U
TUT7	Terminal Uridylyl Transferase 7	ZCCHC6	TENT3B, PAPD6, KIAA1711		In Critical region for Cutaneous Malignant Melanoma Predisposition Locus (Cannon-Albright et al., 2013) Closest ORF to rs952834 Autism susceptibility loci (Weiss et al., 2009)	U
TUT1	Terminal Uridylyl Transferase 1, U6 snRNA-specific	TUT1	PAPD2, STAR-PAP, RBM21	Nucleolus (Trippe et al., 2006) Colocalizes with mRNA cleavage and polyadenylation specificity factor complex (Gonzales et al., 2008) Nuclear Speckles (Mellman et al., 2008)		U and A
TENT2	Terminal Nucleotidyltransferase 2	PAPD4	GLD2, TUT2	Cytoplasm/Nucleus (Nakanishi et al., 2006)		A or A, U, G
TENT4A	Terminal Nucleotidyltransferase 4A	PAPD7	POLS, TRF4-1, POLK, LAK-1	Nucleoplasm > Cytoplasm (Ogami et al., 2013)		A
TENT4B	Terminal Nucleotidyltransferase 4B	PAPD5	TRF4-2	Nucleus/Nucleolar (Lubas et al., 2011; Rammelt et al., 2011; Ogami et al., 2013) Cytoplasm, some cells have additional nuclear staining (Mullen and Marzluff, 2008)		A
MTPAP	Mitochondrial poly(A) polymerase	MTPAP	TENT6, PAPD1, SPAX4	Mitochondrion (Tomecki et al., 2004; Nagaike et al., 2005; Xiao et al., 2006) Nucleus/Cytoplasm/Mitochondrion/Golgi Apparatus (Barbe et al., 2008) Cytoplasm (Mullen and Marzluff, 2008)	spastic ataxia autosomal recessive type 4 (SPAX4) [OMIM:613672] (Crosby et al., 2010)	A

* *Effective date for HUGO-approved change in gene nomenclature was April 12, 2018*.

## Spliceosomal U6 snRNA and uridylation

**Figure 2:**
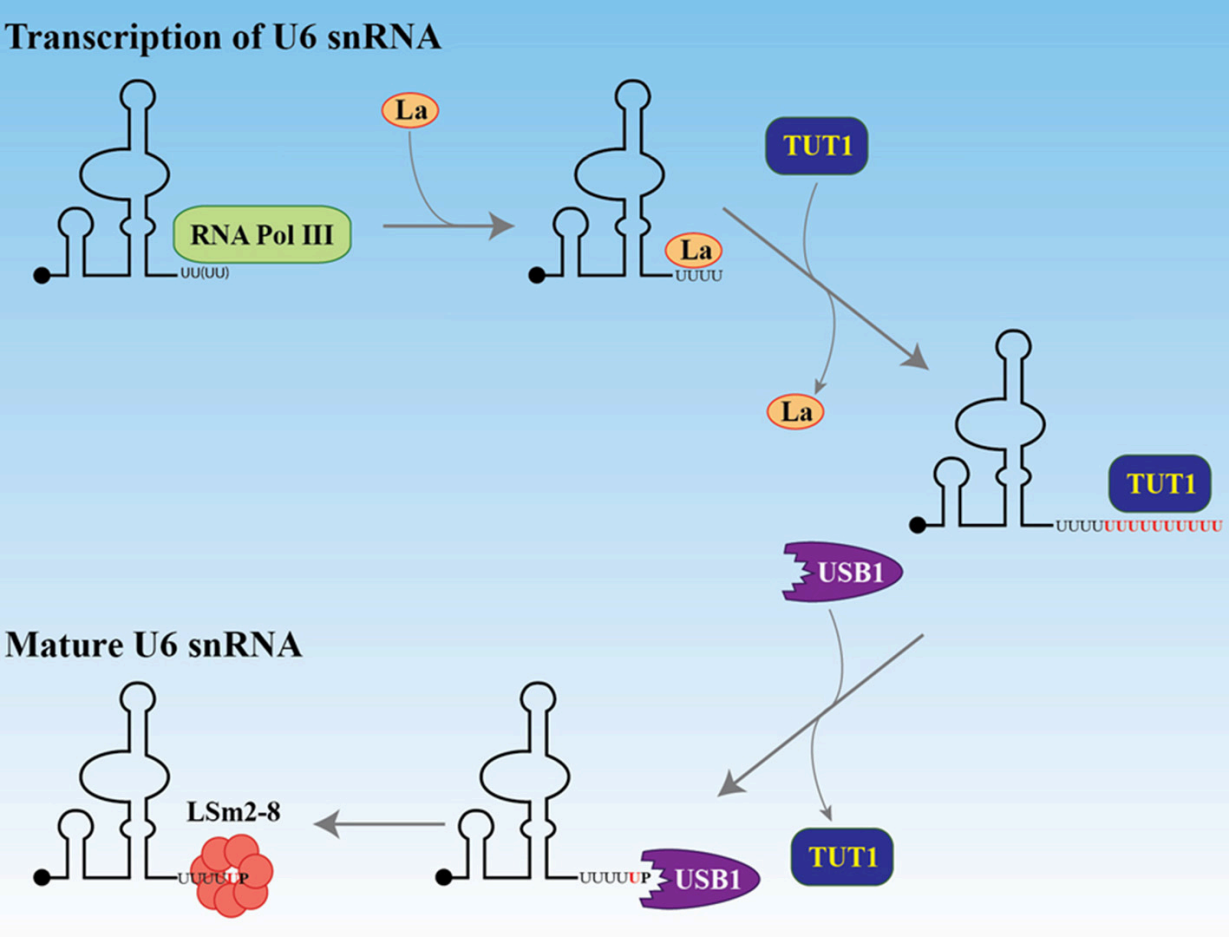
Maturation of U6 snRNA. After transcription by RNA polymerase III, the La protein binds the four uridines at the 3′ end. The black dot at the 5′ end represents the gamma-monomethylguanosine triphosphate cap. The La protein is replaced by TUT1 which adds about 20 uridines to the encoded four. The exonuclease and phosphodiesterase USB1 (MPN1) removes the uridines, keeping five of them and adding a terminal 2′, 3′ cyclic phosphate. This 3′ structure facilitates the recruitment of the LSm2-8 complex and protects the U6 snRNA from degradation by the exosome.

Transcription of eukaryotic protein coding genes, especially in multicellular organisms, generates a pre-mRNA that typically undergoes splicing to remove introns and join successive exons. The major spliceosome, a large ribonucleoprotein (RNP) complex, is required for splicing and contains five essential snRNPs (U1, U2, U4, U5, and U6). U6 small nuclear RNA (U6 snRNA) has a unique maturation mechanism (reviewed in Mroczek et al., 2012). As illustrated in Figure 2, U6 snRNA is the only known RNA substrate where uridylation occurs within the nucleus and this uridylation is essential for its splicing function (Trippe et al., 1998, 2003, 2006). U6 snRNA is transcribed by RNA polymerase III (Kunkel et al., 1986) where transcription termination occurs within a short (~5–6 nucleotide) oligo(dT) DNA stretch. The original U6 snRNA transcript contains four uridines at the 3′ end. After transcription and initial 3′ end formation, the chaperon-like La protein can bind this polyU tail, favoring stabilization of the U6 snRNA (Wolin and Cedervall, 2002). To continue the maturation process, La protein is removed and TUT1 adds up to 20 uridines to the polyU tail. This longer polyU tail is a signal for USB1, a 3′ → 5′ exoribonuclease, to remove uridines, leaving only five of them. USB1 then catalyzes the formation of a 2′,3′-cyclic phosphate at the 3′ end. This maturation process allows the LSm2-8 complex to bind the 3′ extremity, facilitates the proper assembly of mature snRNPs, and is important for nuclear retention (Licht et al., 2008).

## MicroRNAs and uridylation

The following sections describe the biochemical functions that uridylation has in relation to microRNAs, a recently identified class of negative regulators of gene expression. MicroRNA expression is most frequently regulated transcriptionally; however, both microRNA biogenesis as well as generation of isomiRs, sequence variant microRNAs, can be regulated by TUTases. Briefly, uridylation plays both positive and negative roles in regulating canonical microRNA biogenesis for a small, albeit important set of microRNAs, most notably the tumor suppressor let-7 microRNA family. Uridylation is also implicated in two distinct non-canonical microRNA biogenesis pathways that differ on their reliance on Drosha and Dicer. Lastly, uridylation of mature microRNAs can generate isomiRs with altered activity.

### Canonical microRNA biogenesis and function

MicroRNAs are a class of small noncoding RNAs (~19–23 nucleotides) that primarily function as negative regulators of their mRNA targets (reviewed in Hagan and Croce, 2007; Bartel, 2009; Lin and Gregory, 2015b; Balzeau et al., 2017). Mature microRNAs are generated through two step-wise cleavage reactions from the primary microRNA (pri-miRNA) transcripts. Pri-miRNAs are produced by RNA polymerase II (Cai et al., 2004; Lee et al., 2004) and to a much lesser extent by RNA polymerase III (Borchert et al., 2006). The microRNA host gene can be either a protein coding gene or a long non-coding RNA (lncRNA). For protein coding host genes, the microRNA almost exclusively exists in the intron as expected, since microRNA processing would disrupt the mRNA. The few exceptions to this rule are several microRNAs that lie either in alternatively spliced exons or between alternative polyA sites. For host genes that are lncRNAs, microRNA lie in both introns and exons at significant frequencies.

Canonical microRNA biogenesis occurs by two sequential endonucleolytic reactions to produce a mature, functional microRNA. The pri-miRNA is first cleaved by the nuclear RNase III enzyme Drosha in the Microprocessor complex (Lee et al., 2003; Denli et al., 2004; Gregory et al., 2004; Han et al., 2004). The resultant cleavage product is termed precursor microRNA (pre-miRNA) and has a characteristic hairpin structure of ~50–75 nucleotides. The pre-miRNA is transported to the cytoplasm by the Exportin-5/Ran GTP complex (Yi et al., 2003; Bohnsack et al., 2004). In the cytoplasm, the pre-miRNA is processed by a second RNase III enzyme Dicer to release a small RNA duplex that is subsequently loaded onto Argonaute (Bernstein et al., 2001; Hutvagner et al., 2001; Ketting et al., 2001; Chendrimada et al., 2005; Haase et al., 2005). Typically, one strand is preferentially incorporated into the miRISC (microRNA Induced Silencing Complex) where it base pairs with imperfect complementarity to the 3′ UTR of its target mRNAs, resulting in mRNA destabilization and/or translational inhibition. As such, microRNAs may potentially regulate 30% of all mammalian genes (Lewis et al., 2005; Lim et al., 2005).

#### Monouridylation promotes dicer processing of group II precursor microRNAs

Addition of non-templated uridine(s) to the 3′ end of microRNAs is an important post-transcriptional modification that has been shown to impact activity and biogenesis of miRNAs. Specific pre-miRNAs are substrates for monouridylation and/or oligouridylation that can impact microRNA biogenesis positively or negatively, respectively. As the name indicates, monouridylation is the addition of a single non-templated uridine, while oligouridylation and polyuridylation refer to the addition of polyU-tails that can be as long as 30 nucleotides.

**Figure 3:**
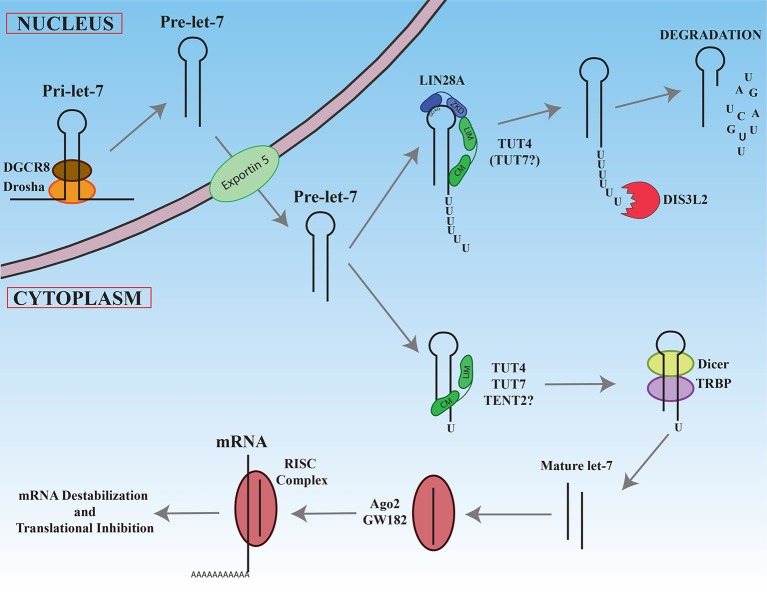
Maturation of let-7 miRNAs. Newly transcribed pri-let-7 is cleaved first in the nucleus by the Drosha/DGCR complex to generate pre-let-7. After export ot the cytoplasm, pre-let-7 can be recognize by the LIN28A protein by its GGAG sequence in the loop. LIN28A recruits via its Zinc knuckle domain (ZKD), TUT4 on its LIN28 interacting module (LIM). The catalytic module (CM) of TUT4 adds a polyU tail to pre-let-7. The resultant this polyU tail blocks Dicer cleavage and is recognized by the exonuclease DIS3L2 that degrades polyuridylated pre-let-7. In the absence of LIN28A, TUT4 and/or TUT7 add only one uridine at the 3′ end, promoting recognition and cleavage by Dicer/TRBP complex. The mature let-7 produced interacts with Ago2, enters in the RISC complex, and base-pairs with its mRNA targets to promote mRNA degradation and/or translational inhibition.

For the vast majority of microRNAs, nuclear Drosha cleavage of the pri-miRNA results in a pre-miRNA with a 2 nucleotide 3′ overhang that is ultimately recognized in the cytoplasm by the Dicer/TRBP complex that directs the second cleavage event (Zhang et al., 2004; Park et al., 2011). As depicted in Figure 3, Narry Kim's lab through sequencing and bioinformatic analyses discovered that a small number of pre-miRNAs are characterized by a single non-templated uridine at the 3′ end that creates a 2 nucleotide overhang (Heo et al., 2012). These atypical precursor microRNAs have been termed Group II and include most let-7 family members (7a-1, 7a-3, 7b, 7d, 7f-1, 7f-2, 7g, 7i, and miR-98) as well as miR-105. Monouridylation of these precursors promotes their subsequent Dicer processing and increases the levels of mature microRNA levels *in vivo*. Sequencing of pre-let-7 revealed that roughly 20% of all pre-let-7 microRNAs were monouridylated at their 3′ ends in HeLa cells that lack expression of both LIN28A and LIN28B. In contrast, all other single nucleotide additions to pre-let-7 combined represent only 1%. Analysis of numerous deep sequencing libraries indicated that the majority of mature Group II let-7 microRNAs are likely processed from monouridylated precursors, as evidenced by their let-7-3p reads. *In vitro* uridylation assays identified three terminal ribonucleotidyl transferases (TUT4/ZCCHC11, TUT7/ZCCHC6, and TENT2/PAPD4/TUT2/GLD2) competent for mono-uridylating pre-let-7a-1 *in vitro* and each had a preference for pre-let-7a-1 with a 1nt vs. 2nt 3′ overhang. The NTP specificity was determined for these enzymes, revealing that TUT4 and TUT7 utilized UTP, while TENT2 could use UTP, GTP, or ATP. *In vivo* knockdowns performed singly or in combination revealed that these enzymes function redundantly in HeLa cells where triple knockdowns resulted in the most significant loss of Group II mature let-7 and mono-uridylated pre-let-7. For single knockdown, TUT7 had the most prominent effect. Similar results were observed for miR-105 in the triple knockdowns. It remains unclear if TENT2 is an *in vivo* TUTase and whether its effects are mediated by monoadenylation rather than monouridylation, given the reported affinity of TENT2 for ATP over UTP (Chung et al., 2016). These results highlight the surprising complexity in how let-7 microRNA biogenesis is controlled either by monouridylation that promotes microRNA maturation versus LIN28A-directed polyuridylation that blocks biogenesis that is described in the next section.

#### Polyuridylation of select precursor microRNAs blocks their biogenesis

To date, the best-characterized example of 3′ RNA polyuridylation blocking microRNA biogenesis involves LIN28A-mediated repression of the let-7 microRNA family (Figure 3) (reviewed in Lee et al., 2016; Balzeau et al., 2017). Briefly, the developmentally regulated RNA binding protein and proto-oncogene Lin28A binds to the terminal loop of pre-let-7 where it recruits the TUT4 to add a short polyU-tail to pre-let-7, blocking Dicer cleavage (Hagan et al., 2009; Heo et al., 2009; Piskounova et al., 2011). Polyuridylated pre-let-7 is rapidly degraded by the exonuclease Dis3L2 that recognizes the polyU-tail (Chang et al., 2013; Ustianenko et al., 2013). Let-7 microRNAs have gained considerable attention due to their prominent roles as tumor suppressors. Notably, loss of let-7 expression and miR-21 overexpression are the most commonly dysregulated microRNAs delineating poor clinical prognosis in cancers (Yang et al., 2010; Wang et al., 2011; Nair et al., 2012). Consistent with this finding, let-7 microRNAs act as tumor suppressors by negatively regulating numerous oncogenes such as MYC, RAS, HMGA2, YAP1, CDK6, CCND1, and BLIMP1. In cancer, reactivation of either LIN28A or LIN28B expression accounts for the widespread repression of the tumor suppressor let-7 microRNA family. The significance of the LIN28/let-7 pathway is further highlighted by the fact that LIN28A is a Thomson reprogramming factor whose expression on its own is necessary and sufficient to make induced pluripotent stem cells (Yu et al., 2007; Buganim et al., 2012).

Let-7 is an evolutionarily ancient microRNA family whose founding member was discovered in *C. elegans* as a heterochronic gene that regulates developmental timing during the transition from late larval to adult cell fates. In humans, there are 12 let-7 family members encoded by eight chromosomes. These microRNAs are quite abundant in most cell types and often account for >10% of the entire cellular microRNA population (Griffiths-Jones et al., 2006). In both embryonic stem cells and many cancer cells, multiple let-7 microRNA primary transcripts are actively generated; however, mature let-7 microRNAs are remarkably low, due to post-transcriptional gene regulation mediated by LIN28 paralogs.

LIN28A and LIN28B are two closely related and developmentally regulated RNA binding proteins and are implicated directly in negatively regulating let-7 microRNA biogenesis (Heo et al., 2008, 2009; Newman et al., 2008; Rybak et al., 2008; Viswanathan et al., 2008; Hagan et al., 2009; Piskounova et al., 2011). These proteins contain two RNA binding domains whose interactions with the loop of immature let-7 is required for high affinity binding. Specifically, an N-terminal cold shock domain binds a stem-loop structure in the pre-E element while the C-terminal Zinc knuckles bind to a conserved GRAG in the 3′ of the loop (Newman et al., 2008; Piskounova et al., 2008; Nam et al., 2011). Let-7 microRNAs negatively regulate both LIN28A and LIN28B mRNAs by direct binding to their 3′ UTRs, thereby establishing a double negative feedback loop and self-enforcing binary switch. Although both LIN28 proteins were initially thought to block Microprocessor cleavage of nuclear pri-let-7, the subcellular localization of these protein suggested an alternate model where LIN28 functions in the cytoplasm to block the conversion of pre-let-7 to its mature and functional form. Reports at the time showed that LIN28A is almost exclusively cytoplasmic (Balzer and Moss, 2007; Polesskaya et al., 2007), while LIN28B exists in the cytoplasm throughout the cell cycle with some nuclear accumulation during S/G2 phases (Guo et al., 2006).

Narry Kim's and our research subsequently discovered that Lin28A recruits TUT4/Zcchc11 that adds an oligouridine tail to the 3′ end of the pre-let-7, blocking Dicer cleavage and provoking the degradation of polyuridylated pre-let-7 (Hagan et al., 2009; Heo et al., 2009). To reach this conclusion, we both used biochemical and cell culture studies to demonstrate that TUT4 is a TUTase and that knockdown of TUT4 elevated mature let-7 levels in Lin28A-expressing cells. Knockdown of no other terminal ribonucleotidyl transferase tested affected mature let-7 levels. Mechanistically, the Zinc Knuckle Domain (ZKD) of LIN28A and the N-terminal region of TUT4 (LIM: LIN28-Interacting Module) is thought to be critical for mediating their protein-protein interactions (Faehnle et al., 2017; Wang et al., 2017). The RNase II exonuclease DIS3L2, a gene whose germline mutation causes Perlman syndrome (Astuti et al., 2012; Higashimoto et al., 2013), has recently been implicated in the degradation of polyuridylated pre-let-7 (Chang et al., 2013; Ustianenko et al., 2013). As expected, Dis3l2 loss does not affect the levels of mature let-7 microRNAs in Lin28A-expressing cells, since polyuridylated pre-let-7 is no longer a viable Dicer substrate.

The Gregory lab has shown that Lin28A and Lin28B *in vitro* can promote uridylation of pre-let-7 by both TUT4 and TUT7, raising the possibility that the LIN28 paralogs can use alternate or redundant TUTases (Thornton et al., 2012). They reported that double knockdown of both TUT4 and TUT7 increased let-7 more than TUT4 knockdown alone in mouse embryonic stem (ES) and P19 embryonal carcinoma cells that express Lin28A (Thornton et al., 2012). Furthermore, this work confirmed earlier studies where TUT7 knockdown on its own does not elevate mature let-7 levels (Hagan et al., 2009; Heo et al., 2009). Our research demonstrated that LIN28A and LIN28B regulate let-7 microRNA biogenesis by distinct mechanisms with differential reliance on the TUT4 (Piskounova et al., 2011). For LIN28B, it remains unresolved the precise mechanism(s) responsible for let-7 regulation, even though in each scenario direct binding of LIN28B to the terminal loop of immature let-7 is involved. Four mechanisms separately or in combination may be important. Specifically, LIN28B may act in the nucleus to block Microprocessor cleavage of pre-let-7, may sequester pri-let-7 in the nucleolus, may block Dicer cleavage of pre-let-7 in the cytoplasm, and lastly, may work with the alternate TUTase TUT7 to block biogenesis. Since the subcellular localization of LIN28B appears cell type dependent, diverse mechanisms of action may be responsible (Guo et al., 2006; Hafner et al., 2010; Piskounova et al., 2011; Molenaar et al., 2012; Suzuki et al., 2015).

In addition to the LIN28A/let-7 pathway, polyuridylation of pre-miRNA has been proposed to repress other microRNAs. Initially, the Kim lab reported that miR-107, miR-143, and miR-200c are regulated by Lin28A and polyuridylation via TUT4 (Heo et al., 2009); however, their followup study argues against this conclusion (Cho et al., 2012). To define RNAs bound to Lin28A, they performed CLIP-Seq, a genome wide method that incorporates UV crosslinking of live cells to capture RNAs bound to a protein of interest that is subsequently enriched via immunoprecipitation. Following protease digestion, high-throughput sequencing of cDNA libraries is performed that correspond to the co-purified bound RNA. Among pre-microRNAs that bind Lin28A, pre-let-7 was discovered but not pre-miR-107, pre-miR-143, or pre-miR-200c. Moreover, knockdown studies confirmed that Lin28A knockdown only upregulates mature let-7 microRNAs, confirming an earlier report (Hagan et al., 2009).

Polyuridylation has also been proposed to regulate miR-1 biogenesis in myotonic dystrophy patients (Rau et al., 2011); however, this work did not report *in vivo* TUTase loss-of-function or gain-of-function experiments. Myotonic dystrophy is caused by expansion of CTG and CCTG repeats in the DMLK and ZNF9 genes, respectively, that create aberrant RNAs that functionally sequester the splicing regulator MBNL1. In heart samples from myotonic dystrophy patients, microRNA profiling revealed that mature miR-1 levels were markedly reduced. In normal cardiac cells, the RNA binding protein MBNL1 binds to the terminal loop of pre-miR-1-1 and pre-miR-1-2 as determined by UV crosslinking. Rau and colleagues hypothesized that in myotonic dystrophy, MBNL1 is sequestered, thereby permitting LIN28 to bind to the pre-miR-1 terminal loop and block miR-1 maturation (Rau et al., 2011). In HeLa cells that do not express either LIN28 paralog, co-transfection experiments showed that LIN28A reduced mature miR-1 when pri-miR-1 was ectopically expressed. *In vitro* uridylation assays showed that Lin28A promotes uridylation of pre-miR-1 by TUT4. Loss-of-function of MBNL1 and Lin28B in H9C2 rat cardiomyoblasts decreased and increased mature miR-1 levels, respectively, consistent with them having antagonistic functions. In myotonic dystrophy patients, miR-1 loss is predicted to cause upregulation of its target genes such as GJA1 (Connexin 43) and CACNA1C (Cav 1.2), leading to dysregulation of important gap junction and calcium channel proteins in the heart.

### Non-canonical microRNA biogenesis

Uridylation also plays roles in non-canonical microRNA biogenesis. In contrast to canonical microRNA biogenesis, a small subset of microRNAs are made by alternative, non-canonical pathways that bypass the requirement for either Drosha or Dicer (reviewed in Miyoshi et al., 2010; Abdelfattah et al., 2014). Dicer-independent and Drosha-independent microRNAs are rare in mammals. The following sections review our current knowledge.

#### Ago2-dependent and dicer-independent microRNAs

For canonical microRNAs, one strand of the microRNA duplex after Dicer cleavage typically is loaded onto an Argonaute protein (Ago1-4), leading to incorporation into the RNA-induced silencing complex (RISC). Although Ago1-4 are individually competent to bind and load microRNAs and siRNAs into RISC, Ago2 is unique in having slicer activity that is responsible for siRNA-mediated (and to a much lesser extent miRNA-directed) cleavage of their target mRNAs.

**Figure 4:**
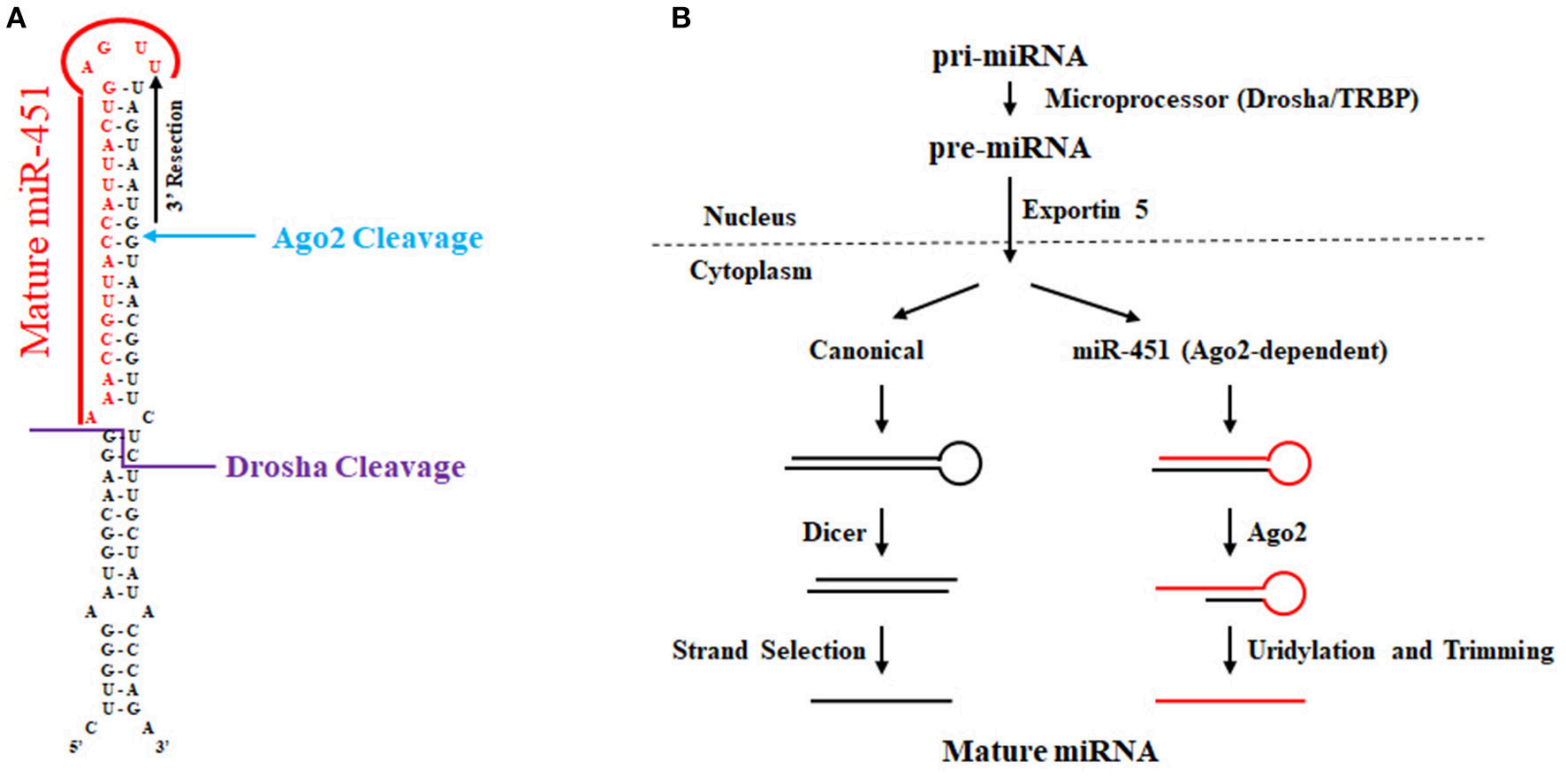
Biogenesis of miR-451. **(A)** Sequence and secondary structure of human pre-miR-451. The location of Drosha and Ago2 cleavage are noted. Uridylation and 3′ trimming ultimately give rise to mature miR-451 as denoted in red font. **(B)** Biogenesis of miR-451 compared to canonical miRNA biogenesis. Compared to the canonical pathway wherein pre-miRNA is processed by Dicer, miR-451 undergoes Ago-2 cleavage that allows for processing to yield mature miR-451 independently of Dicer.

Research initially in zebrafish implicated miR-451 as a key erythropoiesis gene (Dore et al., 2008; Pase et al., 2009) whose function is evolutionarily conserved in mammals (Patrick et al., 2010; Rasmussen et al., 2010). In comparison to other microRNAs as shown in Figure 4, the conserved microRNA miR-451 is rather unusual, since Drosha cleavage yields a short (42 nt) hairpin with only a 17 nucleotide stem that is too short for Dicer cleavage (Siolas et al., 2005). In addition, the mature miR-451 sequence includes the entire hairpin loop. Research using zebrafish and mouse models ultimately led to the discovery that miR-451 biogenesis requires Ago2 rather than Dicer (Cheloufi et al., 2010; Cifuentes et al., 2010). In the zebrafish model, microRNA expression was interrogated in control, maternal-zygotic *dicer* mutants, and maternal-zygotic *ago2* mutants at 48 h post fertilization (Cifuentes et al., 2010). Intriguingly, several microRNAs such as miR-451 and miR-735-5p were unaffected by *dicer* loss. In contrast, mature miR-451 expression was abolished in *ago2* mutants. For miR-451, many reads in wild-type and dicer-deficient zebrafish cells contained the first 30 nucleotides of pre-miR-451 with 1–5 non-templated uridines. The last templated base in this RNA species base-pairs with position 10 in pre-miR-451, a location that is reminiscent of how Ago2 slices mRNA targets using siRNA as a guide in RISC. Further biochemical studies revealed that Ago2 associates with pre-miR-451 and is responsible for pre-miR-451 using *in vitro* processing assays. Their data shows that trimming of Ago2-sliced pre-miR-451 gives rise to mature miR-451. Mouse miR-451 was shown by the Hannon lab to undergo a similar process and was initially discovered using genetically engineered mice where Ago2's slicer activity was abolished by a targeted point mutation that resulted in alanine replacing an essential catalytic aspartate. Their mouse work in conjunction with additional *in vitro* and cell culture assays showed that the biogenesis of miR-451 is Ago2-dependent and Dicer-independent. Recently, another study using the catalytically dead Ago2 mice revealed that miR-486 requires both the catalytic activity of Ago2 as well as Dicer cleavage of its precursor. Specifically, Dicer cleavage of pre-miR-486 produces duplex miRNA-5p/miRNA-3p; however, this duplex is arrested and Ago2 is required to cleave the passenger strand to liberate the guide for its incorporation into the RISC complex.

#### Mirtrons

**Figure 5:**
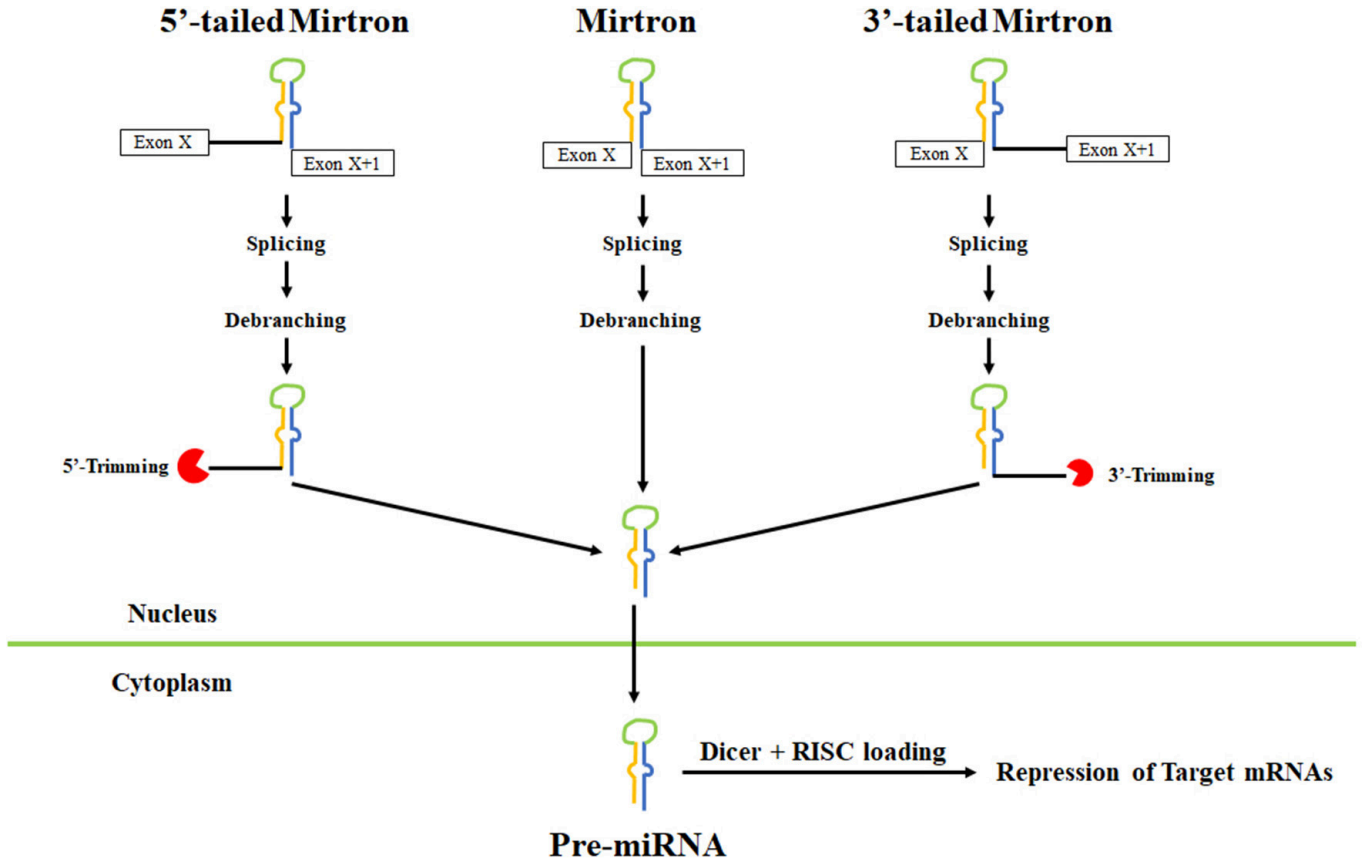
Pathways leading to mirtron biogenesis. Splicing can generate three types of mirtrons. Introns are spliced and the lariat is debranched to allow folding into pre-miRNA-like hairpins. Splicing also leads to tailed mirtrons which undergo additional trimming step after splicing and debranching. For 5′ mirtrons, trimming is carried out by an unknown nuclease while for 3′ mirtrons, trimming is carried out by the exosome. After export to the cytoplasm, the pre-miRNA hairpins are cleaved by Dicer and loaded onto Argonaute complexes.

Another alternative microRNA biogenesis pathway termed “mirtrons” was recently discovered wherein, splicing generates pre-miRNA hairpin mimics, thereby bypassing the requirement for Drosha (reviewed in Rissland, 2015). Mirtrons have been identified in diverse animals including worms, flies, and humans (Berezikov et al., 2007; Okamura et al., 2007; Ruby et al., 2007; Bortolamiol-Becet et al., 2015; Reimao-Pinto et al., 2015). Typical mirtrons have both hairpin ends generated by splicing forming a short 3′ overhang for nuclear export and cleavage (Figure 5). In mammals, short introns are relatively uncommon that could produce a canonical mirtron. A limited number of microRNAs are now definitively known to be mirtrons and include miR-877 and miR-1224 in mammals and miR-1225 and miR-1226 in primates (Berezikov et al., 2007).

Bioinformatic studies have identified hundreds of additional candidate murine and human mirtrons that are predominantly comprised of endogenous Dicer substrates with unique patterns of ordered 5′ and 3′ heterogeneity (Ladewig et al., 2012; Westholm et al., 2012; Wen et al., 2015). In most cases, the end of mammalian mirtrons are characterized by the presence of oligouridine tails due to untemplated uridylation whose functional significance remains unclear. In *Drosophila*, the TUTase Tailor uridylates mirtrons and is linked to mirtron turnover (Bortolamiol-Becet et al., 2015; Reimao-Pinto et al., 2015). It remains unclear how uridylation of mirtrons affects their biogenesis in mammals.

### IsomiRs and uridylation

Mature microRNAs can differ in sequence and length from their canonical miRNA sequence reported in miRBase. These variants, known as isomiRs, are of different types: there is heterogeneity in Drosha and Dicer cleavage site selection, templated additions, or deletions at the 5′ and/or 3′ termini of the miRNA, non-templated additions at the 3′ end and substitutions within the sequence (Morin et al., 2008). Non-templated variations have been attributed to the activity of ribonucleotidyl transferases, posttranscriptional modifications or the presence of genetic variants within the miRNA transcript (Morin et al., 2008; Cloonan et al., 2011; Wyman et al., 2011; Neilsen et al., 2012; Lee et al., 2013; Ma et al., 2013; Starega-Roslan et al., 2015; McCall et al., 2017). IsomiRs due to altered target specificity may regulate different subset of genes compared to the canonical miRNA and may play a role in disease pathogenesis (Gong et al., 2012; Tan et al., 2014). Although this review focuses on uridylation, it is known that other terminal ribouncucleotidyl transferases are significant players in isomiR formation. For example, TENT2 (aka GLD-2) through monoadenylation stabilizes miR-122, a microRNA that is highly expressed in the liver (Katoh et al., 2009; Burns et al., 2011; D'Ambrogio et al., 2012).

To identify and quantify isomiRs, small-RNA-Seq has been routinely used. In this method, sequencing libraries are prepared from size fractionated RNAs to enrich for small RNAs that are used as the initial template. For the high-throughput sequencing itself, a single read is sufficient and the overall number of required reads is less in contrast to mRNA libraries that typically use paired-end reads and require far more sequencing depth. A handful of bioinformatic algorithms exist to analyze small-RNA-Seq data for isomiRs (miraligner, isomiRex, isomiRID, IsomiRage, DeAnnIso, mir-isomiRExp, isomiR-SEA; Pantano et al., 2010; de Oliveira et al., 2013; Sablok et al., 2013; Muller et al., 2014; Guo et al., 2016; Urgese et al., 2016; Zhang et al., 2016). Of interest, a recent report by Garalde and colleagues suggests that direct RNA sequencing using nanopore technology has several beneficial attributes and has the ability to detect specific nucleotide modifications in RNA (Gyarfas et al., 2009). Since the RNA is sequenced directly, nanopore does not lose information relative to post-transcriptional RNA modification in comparison to traditional RNA-Seq. RNA-Seq suffers from additional technical liabilities associated with the library preparation that includes PCR amplification and the short reads make definitive analysis of alternative splicing events difficult.

A striking example of the complexity of post-transcriptional regulation linked to microRNA uridylation involves TUT4, miR-26a, miR-26b, and interleukin-6 (IL-6) (Jones et al., 2009). In LIN28B-expressing A549 cells, Jones and colleagues showed that knockdown of TUT4 decreased the inflammatory cytokine IL-6 at both the mRNA and protein levels. IL-6 mRNA in TUT4 knockdowns had reduced half-life and a shorter polyA-tail length. The IL-6 3′ UTR was sufficient to convey TUT4-dependence on a chimeric luciferase construct. Deep sequencing of A549 cells revealed that miR-26a in control cells exists largely (>80%) as monouridylated 22 nucleotide microRNA, while in TUT4-deficient cells two populations predominate: a 21 nucleotide microRNA missing the 3′ non-templated U (~50%) or as a 23 nucleotide miRNA ending with a non-templated UA dinucleotide (~28%). In contrast, miR-26b reads are increased in TUT4 knockdowns by >20-fold. Luciferase assays showed using microRNA mimics that the ability of miR-26b to repress IL-6 decreases as the length of non-templated Us increase. Overall, their data suggests that TUT4 in part promotes IL-6 expression by decreasing miR-26b levels as well as shifting the isomiR population of miR-26a. A subsequent study has implicated miR-26a in negatively regulating both LIN28B and TUT4, leading to let-7 upregulation (Fu et al., 2013). It remains unknown if TUT4 can regulate directly IL-6 mRNA turnover via microRNA-independent mechanisms.

Recent work from the Gregory and Zon labs have interrogated the consequence of simultaneous knockdowns of both TUT4 and TUT7 on isomiR generation (Thornton et al., 2014). In HeLa cells that do not express the LIN28 paralogs, microRNA sequencing revealed that overall abundance of individual microRNAs was largely unaffected by TUTase loss. Depending on the particular microRNA, up to 8% were determined to be uridylated relative to total reads. TUTase depletion reduced mature microRNA uridylation for multiple microRNAs as expected and resulted in a concomitant gain in adenylation.

## Uridylation in mRNA turnover

Transcription and degradation are both critical for controlling mRNA abundance. Like transcription, mRNA turnover is a regulated process that integrates a multitude of factors including cell-type specificity, timing within the cell cycle, and response to intrinsic and extrinsic signals. Multiple mechanisms exist that are important for mRNA turnover that have differential reliance on deadenylation and uridylation. In the forthcoming sections, we will elaborate on the functions of uridylation in degradation of polyadenylated mRNAs, histone mRNAs that lack polyA-tails, and cleaved mRNAs.

### Uridylation in polyadenylated mRNA degradation

**Figure 6:**
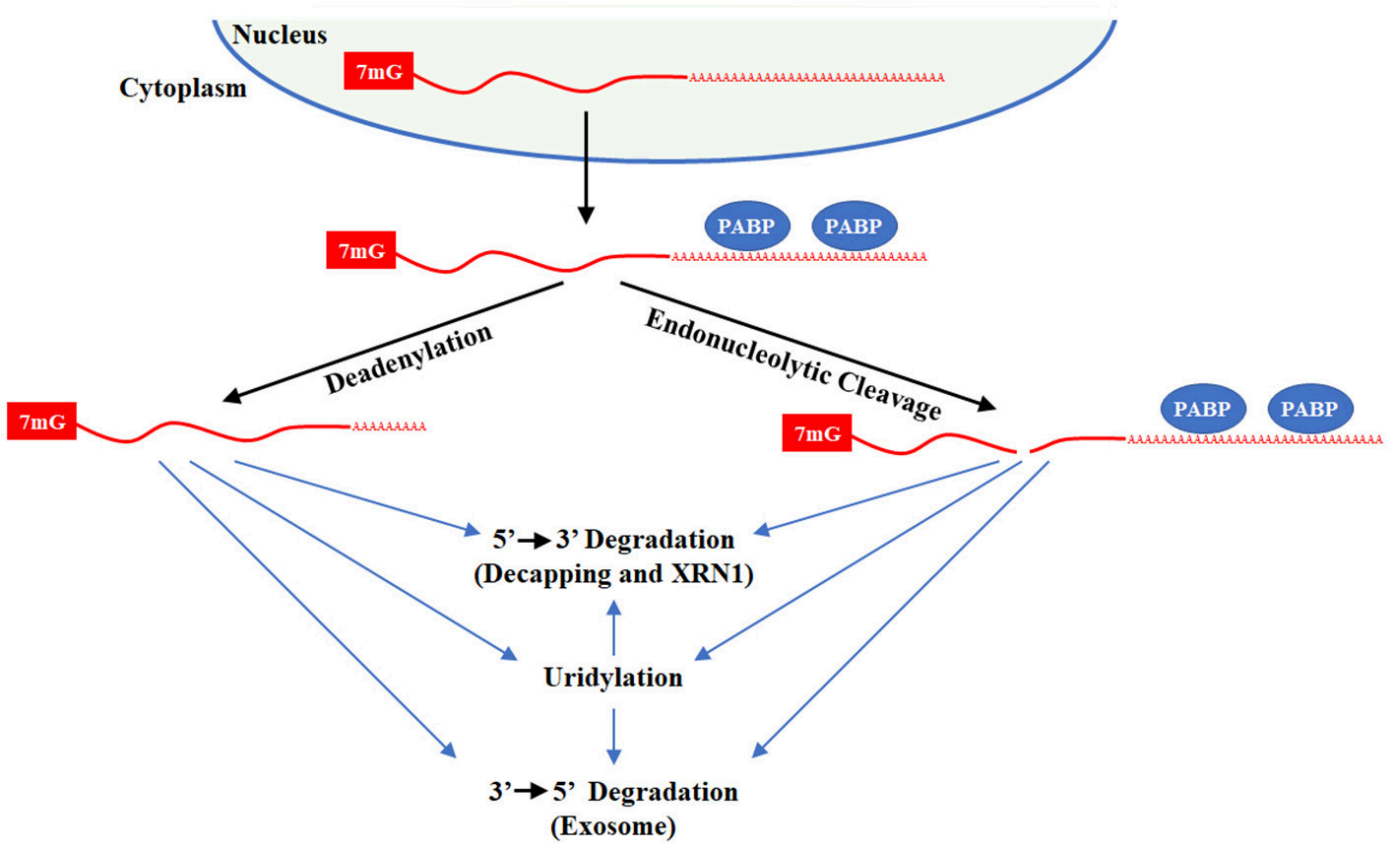
Degradation of polyadenylated mRNAs. Polyadenylated transcripts transit the nucleus into the cytoplasm through the Exportin 5 complex and then binds polyA binding protein (PABP) on its tail. The mRNA can be directly degraded by decapping with DCP1/2 or endonucleolytic cleavage by XRN1 or by the Exosome/DCPS complex. In addition, mRNA deadenylation by CCR4/NOT or PAN2/3 leaves a short poly(A) tail. After deadenylation, the mRNA can be degraded by uridylation-dependent and independent mechanism that rely XRN1/LSM1-7/DCP1/2 or the Exosome/DCPS complex. For uridylated transcripts, TUT7 and/or TUT4 are responsible for uridine addition and their loss stabilizes many mRNAs. DIS3L2 plays a minor role in degradation of uridylated mRNAs since only 1–3 uridines are added to mRNAs with short polyA-tails.

Almost all mRNAs end with a polyA-tail that promotes transcript stability and translational efficiency. Over time, polyA-tails become shorter, decreasing translation and enhancing degradation. Mechanistically, mRNA can be actively degraded by multiple mechanisms where there is significant commonality in the critical enzymes (Figure 6). Decapping and 5′ → 3′ exonuclease, typically XRN1, degrade mRNA from the 5′ end while the exosome degrades mRNA 3′ → 5′. Emerging evidence demonstrates that uridylation contributes to mRNA turnover. The first evidence that uridylation plays a role in mRNA turnover was derived from research on caffeine-induced death suppressor (Cid1) in the fission yeast *Schizosaccharomyces pombe* (Rissland et al., 2007). In *S. pombe*, caffeine in combination with hydroxyurea treatment leads to genomic instability and cell death due to failure in S-M checkpoint control. In a suppressor screen of this phenotype, Cid1 was identified. Bacterially expressed recombinant Cid1 utilizes both UTP and ATP while the Cid1 complex purified for fission yeast acts predominantly as a polyU polymerase that can uridylate multiple mRNAs, targeting them for degradation (Rissland et al., 2007; Rissland and Norbury, 2009).

In mammals, it was widely assumed that polyA tails end in adenine for obvious reasons. In groundbreaking studies by Narry Kim's lab, a novel technique that they termed TAIL-Seq was employed to interrogate the terminal ends of transcripts, revealing that a subset of mRNA transcripts in end U (Chang et al., 2014; Lim et al., 2014). TAIL-Seq is an innovative method to capture the true 3′ end of RNAs. In this method, rRNA are first depleted and a 3′ adapter with biotin is ligated to the 3′ end of the RNA. RNA is fragmented by modest digestion with RNase T1. A streptavidin purification is then used, the 5′ end of RNA is phosphorylated, and a 5′ adapter is added. At this point, library preparation and paired-end high throughput sequencing follows traditional protocols.

Using TAIL-Seq, the Kim lab identified 1–3 uridine addition as a common modification at the end of mRNAs with short polyA-tails (<25 As) and guanylation at the end of mRNAs with longer polyAs (>40 nts) at the downstream of the poly(A) tail. Roughly 50 and 80% of polyadenylated transcripts are uridylated at a frequency of >5 and >2%, respectively, with specific transcript as high as 40%. Considering that uridylation targets mRNAs for degradation, the levels of uridylated transcripts at steady state are rather remarkable. Furthermore, they showed that the TUT4 and TUT7 are the responsible TUTases for mRNA uridylation and their knockdown increased the half-life of numerous transcripts. Overall, they discovered more than >600 genes upregulated in HeLa cells upon TUTase loss. Their data supports the idea that uridylated mRNAs are degraded by canonical pathways where DIS3L2 only has a minor role as anticipated given the limited number of Us added to mRNA. The further importance of TUTases in regulation of the maternal transcriptome during oocyte development and in the maternal-to-zygotic transition that occurs shortly after fertilization is discussed in section TUTases in Vertebrate Development and Physiology.

During apoptosis, uridylation plays a critical role in the rapid degradation of global mRNAs that relies prominently on DIS3L2 (Thomas et al., 2015). For this process, TUT4 and TUT7 are responsible for widespread mRNA uridylation. Knockdown of TUTases or DIS3L2 impaired apoptotic mRNA decay and decreased programmed cell death, while DIS3L2 overexpression promotes apoptosis. When taken together, these results suggest that dysregulated uridylation may play multiple roles in disorders involving apoptotic cell death and/or aberrant mRNA turnover, leading to dysregulated gene expression and cell survival.

### Uridylation and replication-dependent histone mRNA turnover

**Figure 7:**
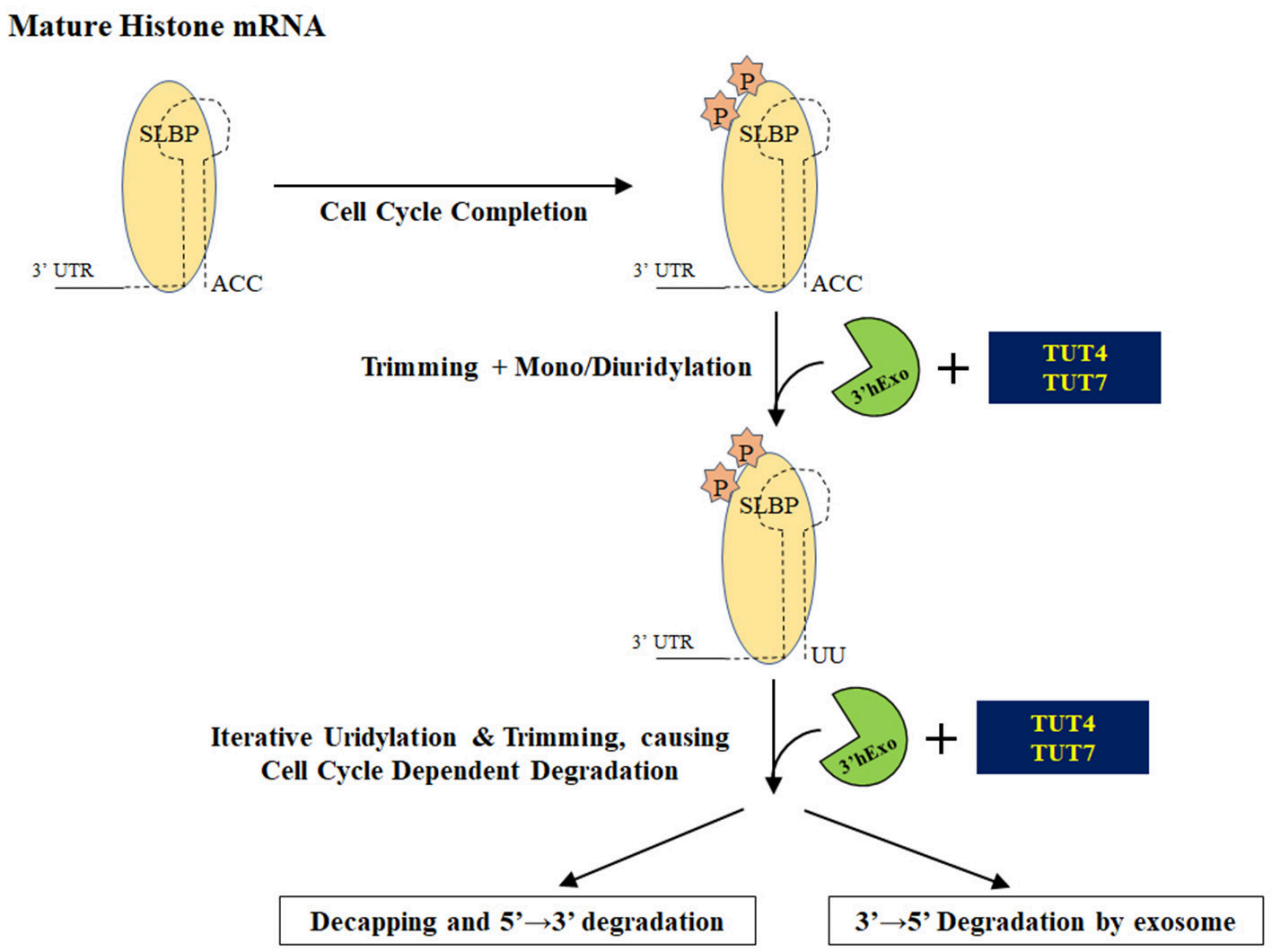
Degradation of histone mRNAs. Upon completion of the cell cycle, stem loop binding protein is phosphorylated and histone mRNAs ending in a stem loop are processed by 3′ hExo. Recruitment of TUTase(s) oligouridylates this structure and initiates degradation of uridylated intermediates either by decapping and/or by the exosome.

Replication-dependent histone mRNAs are exclusively present during the S phase of the cell cycle to encode histone proteins required for packaging of newly synthesized DNA (reviewed in Hoefig and Heissmeyer, 2014; Marzluff and Koreski, 2017). In eukaryotes, this restricted expression is accomplished through regulation of both histone mRNA transcription and degradation (Figure 7). In metazoans, histone mRNAs are unique because they end in a highly conserved stem-loop and are not polyadenylated like other mRNAs. The stem-loop structure interacts with LSm1-7 and Stem-Loop Binding Protein (SLBP) to form a complex that recruits 3′ hExo to bring about the degradation of histone mRNAs at the end of S phase or during the inhibition of DNA replication (Mullen and Marzluff, 2008; Hoefig et al., 2013; Lyons et al., 2014; Brooks et al., 2015).

A pioneering study by Mullen and Marzluff demonstrated that histone mRNA turnover is initiated by 3′ terminal oligouridylation that leads to either 3′ → 5′ degradation by the exosome and/or by decapping and subsequent 5′ → 3′ degradation by the exonuclease Xrn1 (Mullen and Marzluff, 2008). A subsequent collaborative study by the Rhoads and Marzluff labs showed that uridylated histone mRNAs are predominantly degraded by decapping and then 5′ → 3′ degradation (Su et al., 2013). The TUTase(s) involved in oligouridylation of histone mRNAs has not been definitively identified. Different terminal ribonucleotidyl transferases have been proposed as important for histone uridylation: MTPAP, TENT4B, TUT4, and TUT7. The emerging consensus is that TUT4 and/or TUT7 are the responsible TUTase(s). Both MTPAP and TENT4B have been shown to be polyA polymerases and lack the critical histidine that confers UTP specificity (Tomecki et al., 2004; Nagaike et al., 2005; Xiao et al., 2006; Rammelt et al., 2011; Berndt et al., 2012; Boele et al., 2014; Yamashita et al., 2017). Furthermore, MTPAP protein is almost exclusively mitochondrial where it is responsible for polyadenylation on mRNA transcripts encoded by mitochondrial DNA (Tomecki et al., 2004; Nagaike et al., 2005; Xiao et al., 2006). Subsequent work showed that MTPAP likely has an indirect effect on histone mRNA levels, since MTPAP knockdown impairs cell growth and reduces the number of cells actively going through S phase (Su et al., 2013).

In 2011, the Norbury lab showed that knockdown of the cytoplasmic TUT4 in HeLa cells blocked histone mRNA degradation and subsequently stalled DNA replication, accompanied by a proportional reduction in uridylated histone mRNA transcripts (Schmidt et al., 2011). The significance of TUT4 was independently confirmed in a subsequent study (Su et al., 2013). These data suggest that TUT4 is the terminal U-transferase responsible for directing histone mRNAs for degradation upon the inhibition or completion of DNA replication. Lackey and colleagues found through high-throughput sequencing that knockdown of TUT7 reduces uridylation at the 3′ end of histone mRNA transcripts as well as uridylation of degradation intermediates in the stem loop (Lackey et al., 2016). In contrast to prior reports, a knockdown of TUT4 did not produce an effect on the 3′ uridylation pattern or on the stem-loop, suggesting that TUT7 in concert with 3′hExo play a major role in trimming and uridylation of histone mRNAs while TUT4 plays a minor role in this process.

### Uridylation promotes degradation of cleaved mRNAs

The discovery of RNA interference and the experimental utility of shRNA/siRNAs has revolutionized how gene function is interrogated (Mello and Conte, 2004). In cases of perfect (or near perfect) complementarity, the Ago2-RISC complex directs siRNA-directed cleavage of their targeted mRNA. In contrast, miRNAs in the RISC complex due to their imperfect complementarity to their mRNA targets usually result in mRNA destabilization and/or translational inhibition. When Ago2-RISC does cleave the target mRNA, the resulting 5′ mRNA fragment is often characterized by the addition of 1–24 uridines that promotes degradation (Shen and Goodman, 2004).

## Uridylation in non-coding RNA quality control

Non-coding RNAs such as rRNAs, tRNAs, snRNAs, snoRNAs are involved in diverse cellular processes ranging from chromosome replication to mRNA translation. In certain cases, these ncRNAs may be non-functional due to mutations in their genetic sequence, transcriptional errors, premature termination, and misprocessing. Therefore, it is imperative to eliminate aberrant ncRNAs in order to ensure proper cellular function and avoidance of diseases. Elimination of such misprocessed ncRNA precursors is mediated in part by uridylation of ncRNA termini.

Haas and colleagues used a proteomics approach to identify protein complexes involved in miRNA tailing and trimming and identified TUT1 and DIS3L2 as likely candidates to be involved in ncRNA quality control (Haas et al., 2016). Studies with DIS3L2 and its catalytically inactive variant in Perlman syndrome revealed that DIS3L2 recognizes uridylated ncRNAs in the cytoplasm and subsequently degrades them (Labno et al., 2016). In this case, uridylation was carried out by TUT4 and TUT7 regardless of the origin of the ncRNAs. CLiP-Seq studies revealed that DIS3L2 has affinity for various uridylated ncRNAs: unprocessed tRNAs, vault RNAs, Y-YNAs, 5S RNA, snoRNAs transcribed by RNA pol III, transcription start site-associated short RNAs (TSSas), extended snRNAs and a FTL short RNA from the ferritin mRNA 5′ UTR transcribed by RNA polymerase II (Pirouz et al., 2016; Ustianenko et al., 2016). Also, polyuridylation of aberrant precursor snRNAs targets them for degradation by DIS3L2, an exonuclease that recognizes polyU-tails (Faehnle et al., 2014; Labno et al., 2016; Ishikawa et al., 2018). Also, TUT7 and to a lesser extent TUT4 is thought to be critical for elimination of trimmed pre-miRNAs (Kim et al., 2015). These findings suggest that the TUT-DIS3L2 surveillance (TDS) is responsible for monitoring and elimination of aberrantly structured ncRNAs.

## TUTases in vertebrate development and physiology

The *in vivo* functions of TUTases have been elucidated in morphant zebrafish and in genetically engineered knockout mice. In zebrafish, knockdown of either *tut4* or *tut7* via morpholino injection into fertilized eggs causes the majority of fish to die within 5 days post-fertilization (Thornton et al., 2014). These morphant zebrafish are characterized by developmental delay, failure in tail elongation, and somite degeneration. *In situ* hybridization revealed that several Hox genes had aberrant expression patterns in the morphant zebrafish. Of note, the morpholinos used in this experiment block pre-mRNA splicing and therefore do not interrogate the role of the spliced maternal TUTase transcript that exist in the unfertilized egg.

In mammals, TUT4 works with Lin28A to block let-7 microRNA biogenesis (Hagan et al., 2009; Heo et al., 2009; Piskounova et al., 2011), while Lin-28 utilizes the TUTase PUP-2 to suppress let-7 in the nematode *C. elegans* (Lehrbach et al., 2009), implicating uridylation as an ancient mechanism important for Lin28 repression of let-7. Similar to TUTase-deficient fish, loss of either *lin-28a* or *lin-28b* in zebrafish caused multiple phenotypes, including developmental delay (Ouchi et al., 2014). These embryos were characterized by severe gastrulation defects that led to ~40% lethality by 12 h post fertilization and those that survived longer had reduced body lengths and head size.

Knockout mice have elucidated the function of the TUTases TUT4/Zcchc11 and TUT7/Zcchc6 in mammals. TUT7-deficient mice are born in expected Mendelian ratios and appear overtly normal (Kozlowski et al., 2017). TUT4 and TUT7 are broadly expressed at the RNA level where TUT7 is relatively higher in the liver, lungs, and alveolar macrophages. To determine if TUT7 is involved in the defense of inhaled pathogens, wild-type and TUT7 knockout mice were challenged with *S. pneumoniae* through intratracheal exposure. TUT7 nulls had increase neutrophil recruitment during early infection and elevated several cytokine mRNAs such as CXCL5, IL-6, and CXCL1. This finding suggests a role for TUT7 in altering the innate immune response. Organism-wide double knockout of both TUT4 and TUT7 starting at 2 months of age are reported to be overtly healthy for several months, indicating that these genes are likely not essential in adult mammals (Morgan et al., 2017); however, detailed analyses of these mice remain to understand how uridylation may contribute in more subtle ways to adult physiology across multiple tissues.

TUT4 knockout mice are born normal in size and at expected Mendelian ratios (Jones et al., 2012); however, null mice are characterized by postnatal growth retardation that persists throughout life and roughly 50% of nulls die within a week of birth. For knockout mice that survive to weaning age, they are long lived. Reduced levels of insulin-like growth factor 1 (Igf-1) in the liver and the blood were reported to contribute to the observed growth retardation and lethality. Igf-1 is a potent mitogen previously implicated in organismal growth (Baker et al., 1996). Mechanistically, the 3′ UTR of Igf-1 was critical for TUT4-mediated regulation as shown by luciferase reporter assays, suggesting possible microRNA involvement. Of note, deep sequencing of microRNAs in neonatal livers revealed that several mature microRNA species in TUT4-deficient mice had reduced levels of non-templated uridine additions while the overall abundance of individual microRNAs was largely unaffected. For mature microRNAs characterized by differential uridylation, several are predicted to repress Igf-1. The uridylated forms of miR-126-5p and miR-379 have diminished ability to repress luciferase expression constructs that harbor the IGF-1 3′ UTR. Therefore, loss of uridylation of these microRNAs is thought to enhance their ability to repress Igf-1. One caveat to this study is that it was conducted prior to the realization that TUTases promote degradation of mRNAs with short polyA-tails (Chang et al., 2014; Lim et al., 2014).

Regulation of miRNA expression is necessary for CD4 T-cell maturation. Using deep sequencing, Vasquez et al. demonstrated upon T-cell activation, terminally uridylated miRNA sequences decreased, accompanied by a proportional decrease in the levels of TUT4 and TUT7 (Gutierrez-Vazquez et al., 2017). Furthermore, analysis of TUT4 deficient T lymphocytes, demonstrated that TUT4 is essential for the maintenance of miRNA uridylation in steady-state T lymphocytes. This study underscores the role of post-transcriptional uridylation in regulating miRNA levels during T-cell activation.

In mammals, the program for early development is established during oogenesis through the maternal transcriptome which is achieved by stabilization of RNA binding proteins and suppression of RNA degradation pathways. However, during oocyte growth, limited degradation is required to confer distinctiveness to the maternal transcriptome. In a recent study by Morgan et al. TAIL-Seq was used to demonstrate that during oocyte growth in mice, 3′ terminal oligo-uridylation of mRNA that is mediated by TUT4 and TUT7 facilitates the elimination of certain transcripts (Morgan et al., 2017). These events play a critical role in defining the maternal transcriptome. They generated mice in which Zp3-Cre was used to knockout conditionally both TUT4 and TUT7 during oogenesis starting at the secondary stage. TUT4/TUT7 conditional knockout mice were infertile but underwent ovulation normally. Further studies revealed that this phenotype was due to the inability of these animals to support early embryonic development due to meiosis failure. This was mainly attributed due to a lack of elimination of TUT4 uridylated transcripts that resulted in an inaccurate maternal transcriptome. This landmark study revealed that uridylation is imperative for accurate development of the maternal transcriptome, which in turn regulates oocyte maturation and fertility (Morgan et al., 2017).

After fertilization, TUT4 and TUT7 are implicated in the targeted degradation of maternal transcripts during the maternal-to-zygotic transition (MZT) in multiple vertebrate species that include frogs, fish, and mice (Chang et al., 2018). To reach this conclusion, the Kim lab profiled mRNAs by TAIL-Seq at fertilization and immediately thereafter. They discovered that a marked increase in uridylation during MZT that occurs 4–6 h post fertilization. Using TUT4 and TUT7 morpholinos targeting the start codon that blocks translation of the pre-existing spliced maternal transcripts for these two genes, they found that MZT uridylation was impaired, a subset of maternal transcripts was stabilized, and that TUTase loss cause significant gastrulation defects in both zebrafish and frogs. TUT7 was found to be primarily responsible for mRNA uridylation prior to the blastula stage and its loss recapitulated the observed failures in gastrulation. In addition, morpholinos that block splicing of the TUTase pre-mRNAs did not cause MZT defects indicating that new zygotic expression of the TUTases is not required for this process and confirmed the significance of maternal TUTase mRNAs. Altogether, these results highlight that TUTases play critical roles in the elimination of maternal transcript that are required during MZT. It should be noted that TUT7 null mice are born in appropriate Mendelian ratios and as such, its loss in insufficient to cause embryonic lethality (Kozlowski et al., 2017).

To elucidate the developmental roles of Lin28 in mammals and by extension shed light on potential functions for TUTases, we generated and interrogated conditional mouse knockouts for both Lin28A and Lin28B in collaboration with George Daley's group (Zhu et al., 2011; Shinoda et al., 2013a,b). Lin28B nulls are overtly normal except for a modest impairment in male postnatal growth. Lin28A mouse knockouts are born in the expected Mendelian ratios; however, these mice are growth impaired and the vast majority die perinatally. Mechanistically, loss of Lin28A during embryogenesis leads to lifelong defects in glucose metabolism that likely contribute to slowed postnatal growth in survivors. Additional analysis of these conditional knockout as well as gain-of-function Lin28 mice reveal a direct role for Lin28 in regulating glucose metabolism and cellular bioenergetics, in part by let-7 mediated regulation of the Insulin-PI3K-mTOR pathway (Zhu et al., 2010, 2011). Specifically, muscle specific loss of Lin28A or overexpression of let-7 resulted in insulin resistance and impaired glucose tolerance. Double knockout of both Lin28A and Lin28B results in lethality by embryonic day 12.5, with numerous defects including developmental delay and neural tube closure defects, suggesting that the paralogs have partially redundant developmental functions. Conditional knockout of either Lin28A or Lin28B at 6 weeks of age yield no overt phenotypes, demonstrating that these genes do not play essential functions shortly after birth. This result is not surprising as expression of both genes is largely restricted to embryonic development.

## TUTases in disease

Given the relative infancy of the 3′ RNA uridylation field, our understanding of how uridylation contributes to disease is rather limited. Recent and provocative evidence implicates uridylation as a critical gene regulator and driver of tumorigenesis. These data indicate dysregulated TUTase activity alone and in concert with the onco-fetal LIN28/let-7 pathway as hallmarks of poor prognosis in multiple cancer types. In addition, Perlman syndrome and potentially a subset of Wilms tumors are caused by mutations in DIS3L2, a 3′ → 5′ exonuclease that is responsible for degrading numerous polyuridylated RNA substrates. As discussed earlier, polyuridylation may play a role in myotonic dystrophy. As research continues, it is likely that defective uridylation will be discovered that has significance in additional diseases.

### TUTases and the LIN28/let-7 pathway in cancer

The global significance of the onco-fetal LIN28/let-7 pathway has been the subject of multiple reviews, given its importance in ES biology, iPSC reprogramming, and cancer (reviewed in Bussing et al., 2008; Peter, 2009; Viswanathan and Daley, 2010; Lee et al., 2016; Balzeau et al., 2017). The LIN28 paralogs, LIN28A and LIN28B, are RNA binding proteins and proto-oncogenes whose expression occurs primarily during embryonic development. In multiple cancers, one of the LIN28 paralogs will be reactivated, blocking biogenesis of the tumor suppressor let-7 microRNA family. For LIN28A in particular, it recruits the TUT4 to polyuridylate pre-let-7, blocking let-7 maturation and function. Let-7 exerts its tumor suppressor function in part by inhibiting the expression of numerous oncogenes such as MYC, RAS, YAP1, HMGA2, and CDK6 and by altering cellular bioenergetics, including glucose metabolism. LIN28A and LIN28B expression in cancer is typically mutually exclusive and expression of either gene is almost invariably associated with poor prognosis. Since there are 12 let-7 family members encoded by eight human chromosomes, the activation of the LIN28/let-7 pathway explains the global post-transcriptional let-7 repression observed in many human cancers.

The LIN28/let-7 pathway is directly implicated in cancer using genetically engineered mouse models where ectopic LIN28A and/or LIN28B expression cause and/or enhance progression of neuroblastoma, Wilms tumor, mast cell leukemia, hepatocellular carcinoma, and colorectal adenocarcinoma (Molenaar et al., 2012; Nguyen et al., 2014; Urbach et al., 2014; Tu et al., 2015; Wang et al., 2015). Reintroduction of let-7 expression effectively impedes tumor growth or even causes tumor regression in several mouse models (Esquela-Kerscher et al., 2008; Viswanathan et al., 2009; Trang et al., 2010; Piskounova et al., 2011). For example, our research demonstrated that TUT4-depletion or let-7 reintroduction caused established xenograft tumors to regress in mice (Piskounova et al., 2011). LIN28 paralog expression confers resistance via a let-7 dependent mechanism to ionizing radiation and several chemotherapies (Weidhaas et al., 2007; Blower et al., 2008; Yang et al., 2008; Chen et al., 2009; Oh et al., 2010; Boyerinas et al., 2012). Altogether, these data provide strong evidence implicating the onco-fetal LIN28/let-7 pathway in cancer and as an attractive target for new therapies. This view is further reinforced by our conditional mouse knockout studies that demonstrate that loss of either Lin28A or Lin28B proto-oncogenes at 6 weeks of age cause no overt phenotypes (Shinoda et al., 2013b), consistent with predominant embryonic expression of these genes. In addition, conditional knockout of both TUT4 and TUT7 in adult mice are overtly healthy (Morgan et al., 2017). Therefore, targeted inhibition of LIN28 is less likely to cause deleterious side effects in cancer patients. Recently, the Gregory lab has performed a high throughput biochemical screen to identify mouse TUT4 inhibitors (Lin and Gregory, 2015a). Although the majority of initially identified compounds were thiol-containing false positives due to lack of a reducing agent in the screen, several compounds were ultimately identified that could block pre-let-7 uridylation *in vitro*.

In addition to the role of uridylation in LIN28A-expressing cancers, TUT4 is implicated in both gliomas and breast cancer, independently of the LIN28/let-7 pathway. In breast cancer, Hallett and Hassell defined a dual gene classifier to predict breast cancer survival using E2F1 and TUT4 (i.e., KIAA0191; Hallett and Hassell, 2011). Other proliferation-related genes (e.g., BUB1 or AURKA) could substitute for E2F1 in their 2-gene signature and TUT4 was not prognostic on its own. When taken together, these results suggest that TUT4 overexpression in certain contexts may contribute to poor clinical prognosis in breast cancer. Of note, this classifier was independent of breast cancer subtype and the LIN28/let-7 pathway. Proteomic studies have also revealed that overexpression of TUT4 is common in high grade gliomas in comparison to low grade (Gerth et al., 2013). The potential significance of this association remains to be elucidated.

### DIS3L2 in perlman syndrome and wilms tumor

Perlman syndrome is a rare, autosomal recessive congenital overgrowth and cancer susceptibility disorder (Neri et al., 1984, 1985). In 2012, germline mutations in the DIS3L2 exonuclease was discovered by a team working on Perlman syndrome (Astuti et al., 2012). Subsequently, two DIS3L2 heterozygous missense changes were identified in sporadic Wilms tumor, a tumor highly associated Perlman syndrome. Astuti and colleagues showed that the loss of DIS3L2 is associated with mitotic abnormalities and the abnormal expression of mitotic proteins, introducing the tumor suppressor activity of DIS3L2 (Astuti et al., 2012). A case of homozygous deletion of DIS3L2 exon 9 in a Japanese patient with Perlman syndrome was described later (Higashimoto et al., 2013). DIS3L2 is a cytoplasmic RNA exonuclease that is important for degrading numerous polyuridylated non-coding RNAs through recognition of their polyU tails (reviewed in Morris et al., 2013; Pashler et al., 2016).

Recent work has identified multiple non-coding RNAs as direct targets for Dis3l2-mediated decay where the underlying theme is terminal polyuridylation is the key specificity determinant. Chang and colleagues showed that Dis3l2 knockdown in mouse embryonic stem cells does not affect mature let-7 levels, consistent with polyuridylated pre-let-7 no longer being a Dicer substrate; however, an increase in polyuridylated pre-let-7 levels was observed. They normalized their data to U6 snRNA, a key spliceosomal component that undergoes oligouridylation and trimming. *In vitro*, Dis3l2 preferentially interacts with and degrades polyuridylated precursor microRNAs (e.g., pre-miR-21 and pre-let-7g) in comparison to their non-uridylated counterparts, highlighting that a polyU-tail directs at least in part the activity of this enzyme. In the study of Ustianenko and colleagues, they identified Dis3L2 initially as an oligoU binding protein (Ustianenko et al., 2013). They further showed Dis3l2 associates and degrades polyuridylated pre-let-7. Interestingly, in LIN28A- and LIN28B-negative HeLa cells, they found that Dis3L2 loss reduced mature let-7 levels. The potential significance of let-7 microRNAs in Perlman syndrome remains to be resolved. Also, the precise defects that leads to Perlman syndrome is unclear as one can envision that disease is caused by the toxic accumulation of defective RNA species and/or by loss of specific RNA(s) that require DIS3L2 activity for function.

## Future perspectives

Although the last decade has revealed the central importance of uridylation in mammals, much remains to be discovered that has significant implications for basic biology and translational medicine. For example, the LIN28/let-7 pathway is directly implicated in numerous poor prognosis cancers where expression of either LIN28 paralog may confer resistance to ionizing radiation and several frontline chemotherapies such as doxorubicin, 5-fluorouracil, taxanes, and platinum-based drugs (reviewed in Lee et al., 2016; Balzeau et al., 2017). Moreover, cancer stem cells that are often responsible for recurrent and more deadly disease are characterized by LIN28A or LIN28B reactivation even when the tumor bulk lacks their expression. Both the LIN28/let-7 pathway and the TUT4 and TUT7 appear to be *bona fide* molecular targets for therapeutic intervention in cancer where their specific inhibition may be well-tolerated in patients (Piskounova et al., 2011; Shinoda et al., 2013b; Morgan et al., 2017). Altogether, these results suggest that LIN28 and/or TUTase inhibitors may represent a novel drug class whose development is worthwhile.

Further research is also needed to define the precise molecular mechanisms by which TUT4 promotes the development of poor prognosis breast cancers and high grade gliomas, independently of the LIN28/let-7 pathway. Ongoing research continues to unravel how mutations in DIS3L2 contribute to Perlman syndrome and Wilms tumors. Also, advances in next generation sequencing technology such as nanopore sequencing may enhance our ability to look at terminal RNA uridylation that does not involve cumbersome sequencing library construction that is used for small-RNA-Seq or TAIL-Seq. In terms of more basic biology, uridylation is likely regulated in cell-type and disease-specific manners. Therefore, the complete repertoire of functionally significant uridylation targets remains to be elucidated. Numerous biological questions remain about RNA uridylation. What regulates TUTase expression and function? Are there other tissue specific RNA binding proteins that function like LIN28A to target specific microRNAs or other RNA substrates for uridylation-dependent degradation? How functionally significant are isomiRs generated by uridylation in development, normal physiology, and disease biology? What are the precise roles that uridylation plays in non-canonical microRNA biogenesis? Are there cis- and trans-acting factors that modulate uridylation activity on specific RNA substrates such as mRNAs with short polyA-tails? Does dysregulated uridylation contribute to pathology in other diseases and by what mechanisms? The answers to these questions as well as others should provide critical new insights into how uridylation contributes to post-transcriptional gene regulation in the emerging field of RNA epitranscriptomics.

## Author contributions

All authors listed have made a substantial, direct and intellectual contribution to the work, and approved it for publication. MRM and JB contributed equally to this work.

### Conflict of interest statement

The authors declare that the research was conducted in the absence of any commercial or financial relationships that could be construed as a potential conflict of interest.
